# Low physical fitness is a strong predictor of health problems among young men: a follow-up study of 1411 male conscripts

**DOI:** 10.1186/1471-2458-11-590

**Published:** 2011-07-25

**Authors:** Henri Taanila, Antti JM Hemminki, Jaana H Suni, Harri Pihlajamäki, Jari Parkkari

**Affiliations:** 1 Tampere Research Centre of Sports Medicine, the UKK Institute, PO Box 30, 33501 Tampere, Finland; 2 Research Department, Centre for Military Medicine, Lahti and Helsinki, Finland; 3 Research Unit of Pirkanmaa Hospital District and Department of Trauma, Musculoskeletal Surgery and Rehabilitation, Tampere University Hospital, Tampere, Finland

**Keywords:** epidemiology, exercise, fitness testing, sporting injuries

## Abstract

**Background:**

Military service in Finland is compulsory for male citizens and annually about 90% of 19-year-old men enter into the service. Approximately 15% of them are discharged due to medical reasons constituting a group of young men who are at risk of being marginalised in society. The purpose of the study was to evaluate predictive associations between medical discharge from the compulsory military service and various intrinsic risk factors, including socio-economic, health, health behavior, and physical fitness outcomes.

**Methods:**

We followed four successive cohorts of conscripts who formed a representative sample of Finnish young men (18-28 years old, median age 19 yrs) for 6 months. To exclude injuries and illnesses originating before the onset of service, conscripts discharged from the service at the medical screenings during the 2-week run-in period were excluded from the analyses. Data regarding medical discharge were charted from computerised patient records. Predictive associations between medical discharge and intrinsic risk factors were examined using multivariate Cox's proportional hazard models.

**Results:**

Of 1411 participants, 9.4% (n = 133) were discharged prematurely for medical reasons, mainly musculoskeletal (44%, n = 59) and mental and behavioral (29%, n = 39) disorders. Low levels of physical fitness assessed with a 12-min running test (hazard ratio [HR] 3.3; 95% confidence interval [CI]: 1.7-6.4), poor school success (HR 4.6; 95% CI: 2.0-11.0), poor self-assessed health (HR 2.8; 95% CI: 1.6-5.2), and not belonging to a sports club (HR 4.9; 95% CI: 1.2-11.6) were most strongly associated with medical discharge in a graded manner. The present results highlight the need for an improved pre-enlistment examination and provide a new means of identifying young persons with a high risk for discharge.

**Conclusions:**

The majority of the observed risk factors are modifiable. Thus preventive measures and programs could be implemented. The findings suggest that increasing both aerobic and muscular fitness is a desirable goal in a pre-training program before entering military service. Attention to appropriate waist circumference and strategies addressing psychological well-being may strengthen the preventive program. Optimally the effectiveness of these programs should be tested in randomized controlled intervention studies.

## Background

Military service in Finland is compulsory for all male citizens over 18 years of age and the duration varies from 6 to 12 months. The last stage to easily contact an entire age cohort of young males in Finland is at the time of military call-up at 18 years of age. Therefore, a call-up with a medical examination offers a unique opportunity to identify those persons requiring special attention [1]. Approximately 13% to 15% of Finnish conscripts (3500-4000 persons annually) are prematurely discharged from military service for medical reasons [2]. Given that 90% of young men in Finland enter into military service, the high number of medical-related discharges is a public health concern [3].

It is important for military forces to identify persons unsuitable for service as early as possible [4,5], preferably at call-up before entering the service [1]. Early discharge from compulsory military service is a major drain of financial resources and time [6,7]. For the young individual, early discharge during military service can cause financial, emotional, and physical harm [1,8]. Moreover, severe injuries may result in functional impairment that leads to disabilities requiring long-term rehabilitation [9].

Knapik and colleagues [6] reported that lower performance in army physical fitness tests, lower educational level, and injuries accounting for time lost from service are risk factors for discharge in United States Army recruits, consistent with previous findings [8,10,11]. Other risk factors for discharge identified foremost in professional armies include: female sex [4,6,12], older age [7,12], Caucasian race [6,8], tobacco smoking [5,10,13,14], no history of competitive exercise [7], recurrent back pain prior to entering the service [4], history of depression [4,15,16], misconduct [5,12], lack of motivation [15], pre-service injuries [17,18] especially those with incomplete recovery [7,14], poor self-rated physical fitness on arrival [7,14], and low pre-service physical activity [12,14]. Physical and mental problems often overlap, leading to premature discharge from military service [12,18]. Moreover, some researchers have suggested that it is better to focus on overall discharge when examining the value of screening methods [4,5].

The findings from recruit armies are not directly comparable with those of a conscription army. The number of recruits, their quality and motivation, as well as practices and training regimens differ substantially between conscription and hired armies [8,9]. A recent Finnish study focusing mainly on psychological risk factors concluded that men prematurely discharged from compulsory military service require psychosocial support due to the accumulation of mental and social problems [19]. They are at risk of being marginalised in society at a time when they are at the threshold of adulthood [1,20]. In addition to Finnish studies [1,16,19], only one study has investigated risk factors for premature discharge in a conscription army. In Sweden, Larsson et al. [14] found a strong association between musculoskeletal injuries or complaints and discharge. These findings cannot be generalised, because less than 6% of young men complete their military service in Sweden [19].

The purpose of the present 6-month prospective follow-up study of four successive cohorts conscripted in the Finnish army was to evaluate predictive associations between medical discharge of the conscripts and various intrinsic risk factors, including socio-economic, health, health behaviour, and physical fitness outcomes. We hypothesized that low levels of physical fitness and health-damaging behaviour at the beginning of military service are associated with an increased incidence of premature discharge from military training.

## Methods

### Subjects

**Figure 1:**
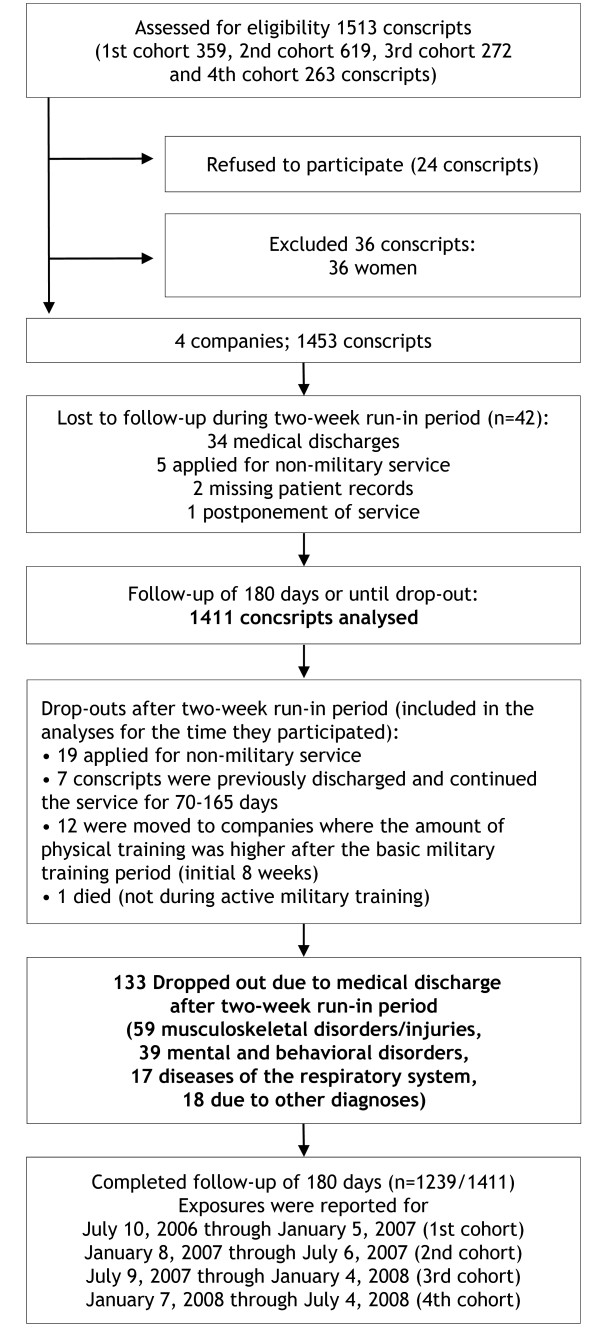
**Flow of conscripts through the study**.

The subjects of the study comprised male conscripts (*N *= 1513) from four companies of one brigade (Pori Brigade, Säkylä) in the Finnish Defence Forces. The companies enrolled into the study were anti-tank, signal, mortar and engineer companies. During the study period, four cohorts of conscripts started service in the brigade (Figure 1). The Pori Brigade is a typical Finnish garrison and the selected companies form a representative sample of conscripts. The conscripts of each age-cohort are randomly assigned into the companies. The baseline characteristics of the companies are presented in Table 1.

**Table 1:** Baseline characteristics of 1411 male conscripts by company.

Variable	Anti-tank	Signal	Mortar	Engineer	Missing	P-value^1^
Number of conscripts	263	540	363	245	0 (0%)	-
Age, median, years (SD)	19 (0.79)	19 (1.18)	19 (0.78)	19 (0.93)	0 (0%)	0.422 ^2^
Body mass index, median, kg/m^2 ^(SD)	23.4 (3.95)	22.6 (3.81)	23.3 (4.17)	23.6 (3.99)	139 (10%)	0.003 ^2^
Waist circumference, median, cm (SD)	87.0 (10.2)	84.9 (9.69)	85.6 (10.5)	87.0 (9.72)	101 (7%)	0.001 ^2^
12-minute run test result, median, m (SD)	2310 (338)	2308 (341)	2500 (302)	2400 (303)	42 (3%)	< 0.001 ^2^
Conscript's physical fitness index (CPFI)^4^, median, points (SD)	15.05 (3.05)	14.75 (3.29)	17.00 (3.10)	15.50 (3.09)	46 (3%)	< 0.001 ^2^
Hometown population ≥ 10,000 persons,%	59%	66%	59%	57%	24 (2%)	0.044 ^3^
High level of education^5^,%	48%	38%	49%	35%	22 (2%)	< 0.001 ^3^
High level of previous physical activity^6^,%	31%	24%	42%	36%	23 (2%)	< 0.001 ^3^
Good self-assessed health^7^,%	57%	47%	61%	51%	22 (2%)	< 0.001 ^3^
Chronic impairment or disability,%	17%	15%	16%	17%	27 (2%)	0.802 ^3^
Regular medication, %	10%	13%	11%	8%	26 (2%)	0.220 ^3^
Clear musculoskeletal symptoms^8^,%	28%	32%	26%	27%	23 (2%)	0.339 ^3^
Previous or current regular smoker, %	43%	47%	44%	57%	26 (2%)	0.004 ^3^
Use of alcohol ≥ 3 times per week, %	16%	19%	15%	20%	23 (2%)	0.318 ^3^

SD = standard deviation

^1 ^P-value for difference between the companies.

^2 ^P-value was calculated using a Kruskal-Wallis test for median difference.

^3 ^P-value was calculated using χ2 statistics for difference.

^4 ^CPFI = (12-min running test result (metres) + 100 × muscle fitness test points)/200, [Excellent (CPFI ≥ 21.00), Good (17.00 ≤ CPFI < 21.00), Fair (13.00 ≤ CPFI < 17.00), Poor (CPFI < 13.00)].

^5 ^Graduated or studies in higher education institution.

^6 ^Sweating exercise at least three times per week during the last month before entering the military.

^7 ^Compared to age-mates.

^8 ^Symptoms lasting more than 7 days in at least one anatomical region during the last month before entering the military.

Twenty-four conscripts (< 2%) refused to participate in the study (Figure 1). All of the remaining conscripts (*N *= 1489) agreed to participate and provided their informed consent before initiation of the study. Because there were only 36 women who volunteered military service and participated in the study (2.4%), their data was excluded from the analysis. Conscripts entering military service were young healthy men, all of whom had a medical check-up by a clinician during the 12 months before entering into the military. The health status of the conscripts was rechecked at baseline using routine medical screenings performed by a physician. To exclude injuries and illnesses originating before the onset of military service, conscripts discharged from the service at the medical screenings during the 2-week run-in period were excluded from the analyses leaving 1411 conscripts included in the analyses (Figure 1).

The age of the conscripts ranged from 18 to 28 years (median 19). All subjects were planned to be followed for 6 months beginning on the first day of service, but some dropped-out from the military or changed company (Figure 1) and this was taken into account when calculating exposure times. Approval for the study protocol was obtained from the Ethics Committee of Pirkanmaa Hospital District on 11 April 2006.

### Physical training program

At the beginning of military service, all conscripts performed 8 weeks of basic training consisting of varying physical activities including marching, cycling, skiing, orienteering, swimming, drill training and combat training, or other training. There was an average of 17 hours of military actions per week with a gradual increase in intensity. Most of this time was low-to-moderate intensity activity. Instructors of the companies supervised that the intensity of training was low-to-moderate level. The rest breaks were organized in such manner that all conscripts managed to perform physical training regularly. In addition, conscripts performed other physical exercises such as jogging, team sports, and circuit training, for an average of 7 hours per week.

The two month basic training period was followed by a specific military training program depending on the company and service duration. During this 4-month period of service, the amount and intensity of physical training was maintained at approximately the same level in different companies.

### Discharge registration and outcome definition

The data were collected from July 10, 2006 to July 4, 2008 (Figure 1). Data regarding medical discharge were charted from computerised patient records. During military service, all conscripts were required to use the services of the military healthcare units. In addition, we received separate discharge statistics from the Pori Brigade and cross-checked this data with the patient records to ensure that the data were complete. Discharges were divided into four main categories according to International Statistical Classification of Diseases and Related Health Problems (10th Revision): musculoskeletal disorders and injuries (M- and S-diagnoses), mental and behavioural disorders (F-diagnoses), respiratory diseases (J-diagnoses), and other diagnoses (Table 2). Discharge from military service was indicated when a physician determined a conscript unable to continue military training.

**Table 2:** Numbers and reasons for early medical discharge from military service after the 2-week run-in period in 1411 male conscripts during a 6-month military training period.

Number	Diagnosis
**Musculoskeletal disorders & injuries**	
25	Overuse injury of the limb
9	Low back pain
8	Internal injury of the knee joint
4	Dislocations
3	Fracture of neck of femur
2	Other chest pain due to earlier fracture
2	Fracture of humerus
1	Fracture of carpal bones
1	Injury of the extensor muscle and tendon of a finger
1	Fracture of shaft of femur
1	Sprain of collateral ligament of knee
1	Sprain of wrist
1	Tendinopathies
**Total 59 conscripts, 44% of all discharges**	
	
**Mental and behavioural disorders**	
21	Adjustment disorders
9	Depressive episodes
7	Anxiety disorders
2	Personality disorders
**Total 39 conscripts, 29% of all discharges**	
	
**Diseases of the respiratory system**	
9	Acute upper respiratory infection
6	Asthma
1	Chronic pansinusitis
1	Chlamydial pneumonia
**Total 17 conscripts, 13% of all discharges**	
	
**Dermatological diseases**	
1	Atopic dermatitis
1	Erysipelas
1	Allergic urticaria
1	Pilonidal cyst without abscess
**Total 4 conscripts, 3% of all discharges**	
	
**Cardiovascular disorders**	
1	Tachycardia
1	Subarachnoid haemorrhage
**Total 2 conscripts, 2% of all discharges**	
	
**Gastrointestinal diseases**	
1	Ulcerative colitis
1	Volvulus
**Total 2 conscripts, 2% of all discharges**	
	
**Other reasons**	
1	Hematuria
1	Postviral fatigue syndrome
1	Allergy unspecified
1	Noise effects on inner ear
1	Precordial pain
1	Malaise and fatigue
1	Congenital pes planus
1	Coma unspecified
1	Acute atopic conjunctivitis
1	Juvenile rheumatoid arthritis
**Total 10 conscripts, 8% of all discharges**	

### Assessment of physical fitness

A Cooper's test (12-min running test) and muscular fitness tests were performed by most (97%) conscripts during their first 2 weeks of military service. A minority of conscripts (3%) were unable to complete their physical fitness tests due to minor health problems, such as infection or overuse injury. Muscular fitness tests included push-ups, sit-ups, pull-ups, the standing long jump, and a back-lift test. Instructors of the companies supervised the conscripts to ensure technically correct performance of each test. More detailed information about the physical fitness tests was presented in our previous study [9].

A poor result in an individual muscle fitness test equated to 0 points, a fair result to 1 point, a good result to 2 points, and an excellent result to 3 points. A conscript's physical fitness index (CPFI) was calculated using the following formula: (12-min running test result [metres] + 100 × Muscle fitness test points)/200. The formula is based on standard practice in the Finnish Defence Forces since 1982 [21]. Because excellent results in the Cooper's test were uncommon (< 4%), the two highest levels, good and excellent, were combined to obtain a group of equal size for comparison between different fitness categories. Individual muscle fitness test results were combined into a single variable to explore whether the combined fitness variable, representing co-impairment, would be more strongly associated with premature discharge. In addition, height, weight, and waist circumference were measured during the first 2 weeks of service. Body mass index (BMI) was calculated by dividing weight (kilograms) by the square of height (meters). Waist circumference, as a mark of abdominal obesity and excessive visceral fat [22], was measured with a tape at the midway between the lowest rib and iliac crest after normal exhalation. The cut-off points to describe overweight and obesity for BMI and waist circumference were set according to the World Health Organisation [23].

### Pre-information questionnaire

Subjects were administered a pre-information questionnaire during the first week of military service. Questions charted conscripts' *socio-economic factors *(Table 3), *health *(Table 4), and *health behaviour *(Table 5) at the baseline of the study. The socio-economic factors included education, urbanization level of the place of residence, educational level, degrees achieved in school, and father's occupational group. Health factors included previous sports injuries and orthopedic surgeries, medication, chronic disease, chronic impairment or disability, self-assessed health compared to age mates, and musculoskeletal pain in six anatomical regions during the last month. Health behaviour was assessed with questions on the use of alcohol and tobacco, frequency of drunkenness, amount of physical exercise, prior sporting activities, belonging to a sports club, participation in competitive sports, highest level achieved in school sports, self-assessed physical fitness, and opinion about the physical demands of a soldier.

**Table 3:** Hazard ratios (HR) for early medical discharge from military service by socioeconomic variables at baseline.

Socioeconomic background & company	Category	Total number (% of discharged)	HR for discharge (n = 133) *	HR for discharge (n = 133) **
Father's occupation	Not physical	488 (8)	1 (Referent)	1 (Referent)
	Physical	590 (10)	1.2 (0.8-1.9)	1.0 (0.7-1.6)
	Unclear or unemployed	261 (10)	1.3 (0.8-2.2)	1.2 (0.7-2.0)
				
School success	Excellent ^1^	218 (4)	1 (Referent)	1 (Referent)
(educational level and	Good ^2^	608 (8)	**2.2 (1.0-4.7)**	2.0 (0.9-4.2)
grades combined)	Satisfactory ^3^	467 (11)	**3.2 (1.5-6.7)**	**2.5 (1.2-5.5)**
	Poor ^4^	96 (22)	**6.4 (2.8-14.5)**	**4.6 (2.0-11.0)**
				
Level of education	High ^5^	589 (6)	1 (Referent)	1 (Referent)
	Lower ^6^	800 (12)	**2.0 (1.4-3.0)**	1.3 (0.7-2.4)
				
Degrees achieved in	High	466 (6)	1 (Referent)	1 (Referent)
school	Low or average	922 (11)	**1.7 (1.1-2.5)**	0.8 (0.5-1.4)
				
Urbanisation level of	< 10000 inhabitants	537 (7)	1 (Referent)	1 (Referent)
the place of residence	≥ 10000 inhabitants	850 (11)	1.4 (1.0-2.0)	1.4 (1.0-2.1)
				
Age	18-19 years	1052 (8)	1 (Referent)	1 (Referent)
	20-28 years	359 (13)	**1.6 (1.1-2.3)**	1.4 (0.9-2.0)
				
Company	Anti-tank company	263 (7)	1 (Referent)	1 (Referent)
	Signal company	540 (10)	1.5 (0.9-2.6)	1.2 (0.7-2.1)
	Mortar company	363 (11)	1.7 (1.0-2.9)	1.7 (0.9-3.0)
	Engineer company	245 (9)	1.2 (0.6-2.3)	1.1 (0.6-2.1)

Variable distribution was charted in 1411 male conscripts during the first week of military service and discharge outcomes were registered during the following 6-month military service. Statistically significant findings are indicated with bold type.

^1 ^Attended upper secondary school, polytechnic, or university and reported excellent or good grades.

^2 ^Other subjects from upper secondary school, polytechnic, or university and conscripts from vocational school whose grades were excellent or good.

^3 ^Respondents with poorer grades in vocational school.

^4 ^Attended only comprehensive school or had permanently interrupted vocational or upper elementary school.

^5 ^Secondary school graduates, polytechnic, and university students

^6 ^Only comprehensive or vocational school

* Adjusted for age (univariate)

** Adjusted for age, company, smoking (previous or current smoker), alcohol intake, baseline medical conditions (sports injury during last month, sum factor of earlier musculoskeletal symptoms during the last month before entering the military, chronic impairment or disability due to prior musculoskeletal injury, chronic disease, regular medication), school success (educational level and grades combined), urbanisation level of the place of residence, participating in ball games, last degree achieved in school sports, physical activity during the previous 3 months before entering the military, self-assessed health, belonging to a sports club and participation in competitive sports (17 adjusting variables).

**Table 4:** Hazard ratios (HR) for early medical discharge from military service by health variables at baseline.

Health variable	Category	Total number (% of discharged)	HR for discharge (n = 133) *	HR for discharge (n = 133) **
Body mass index	Underweight (BMI < 18.5)	56 (7)	1.4 (0.5-3.9)	1.3 (0.5-3.8)
(BMI = (kg)/(m)^2^)	Normal (18.5 ≤ BMI < 25.0)	812 (5)	1 (Referent)	1 (Referent)
	Pre-obese (25.0 ≤ BMI < 30.0)	300 (6)	1.1 (0.6-1.9)	1.1 (0.6-2.0)
	Obese (BMI ≥ 30.0)	104 (9)	1.7 (0.8-3.4)	1.7 (0.8-3.6)
				
Waist circumference	Thin (WC < 80)	271 (7)	1.5 (0.9-2.6)	1.2 (0.7-2.2)
(WC, cm)	Normal (80 ≤ WC < 94)	739 (5)	1 (Referent)	1 (Referent)
	Increased (94 ≤ WC < 102)	178 (6)	1.1 (0.5-2.2)	0.9 (0.4-1.9)
	High (WC ≥ 102)	122 (12)	**2.5 (1.4-4.5)**	**2.4 (1.3-4.6)**
				
Height (cm)	Shortest tertile (≤ 177 cm)	392 (6)	1.3 (0.7-2.3)	1.3 (0.7-2.3)
	Middle tertile (178-183 cm)	477 (6)	1.2 (0.7-2.2)	1.2 (0.7-2.2)
	Tallest tertile (≥ 184 cm)	403 (5)	1 (Referent)	1 (Referent)
				
Self-assessed health ^1^	Good or very good	743 (5)	1 (Referent)	1 (Referent)
	Average	558 (12)	**2.4 (1.6-3.5)**	**1.7 (1.1-2.6)**
	Inferior	88 (26)	**5.7 (3.4-9.5)**	**2.8 (1.6-5.2)**
				
Chronic disease	No	1012 (8)	1 (Referent)	1 (Referent)
	Yes	377 (14)	**1.8 (1.3-2.6)**	**1.6 (1.1-2.3)**
				
Regular medication	No	1235 (9)	1 (Referent)	1 (Referent)
	Yes	150 (15)	**1.8 (1.2-2.8)**	1.3 (0.8-2.2)
				
Orthopaedic surgery	Never	1273 (10)	1 (Referent)	1 (Referent)
	Yes	114 (7)	0.7 (0.3-1.4)	0.8 (0.4-1.7)
				
Chronic impairment	No	1165 (9)	1 (Referent)	1 (Referent)
or disability ^2^	Yes	219 (13)	**1.5 (1.0-2.3)**	1.1 (0.7-1.8)
				
Sports injury during	No	1254 (9)	1 (Referent)	1 (Referent)
last month	Yes	130 (15)	**1.7 (1.0-2.7)**	**1.7 (1.0-2.9)**
				
Sum factor of other	Minimal symptoms ^3^	440 (6)	1 (Referent)	1 (Referent)
musculoskeletal	Mild symptoms ^4^	548 (9)	1.5 (0.9-2.4)	1.3 (0.8-2.2)
symptoms	Clear symptoms ^5^	400 (13)	**2.3 (1.4-3.6)**	1.6 (1.0-2.9)

Variable distribution was charted in 1411 male conscripts during the first week of military service and discharge outcomes were registered during the following 6-month military service. Statistically significant findings are indicated with bold type.

^1 ^Compared to age-mates

^2 ^Due to prior musculoskeletal injury.

^3 ^'Minimal symptoms': maximum 7-day lasting symptom in one anatomical region during the last month before military entry.

^4 ^'Mild symptoms': symptoms in 2 to 6 anatomical regions but the symptoms had lasted a week maximum during the last month before military entry.

^5 ^'Clear symptoms': included the remaining conscripts.

* Adjusted for age (univariate)

** Adjusted for age, company, smoking (previous or current smoker), alcohol intake, baseline medical conditions (sports injury during last month, sum factor of earlier musculoskeletal symptoms during the last month before entering the military, chronic impairment or disability due to prior musculoskeletal injury, chronic disease, regular medication), school success (educational level and grades combined), urbanisation level of the place of residence, participating in ball games, last degree achieved in school sports, physical activity during the previous 3 months before entering the military, self-assessed health, belonging to a sports club and participation in competitive sports (17 adjusting variables).

**Table 5:** Hazard ratios (HR) for early medical discharge from military service by health behaviour variables at baseline.

Health behaviour	Category	Total number (% of discharged)	HR for discharge (n = 133) *	HR for discharge (n = 133) **
Smoking habits	Never smoked regularly	735 (7)	1 (Referent)	1 (Referent)
	Has smoked regularly	650 (12)	**1.6 (1.2-2.3)**	1.3 (0.8-1.9)
				
Use of alcohol	< 1 time per month	254 (13)	1 (Referent)	1 (Referent)
	1-2 times per week	894 (8)	**0.6 (0.4-0.9)**	**0.5 (0.3-0.8)**
	≥ 3 times per week	240 (11)	0.8 (0.5-1.4)	**0.5 (0.3-1.0)**
				
Frequency of drunkenness	< 1 time per week	1075 (9)	1 (Referent)	1 (Referent)
before military service	≥ 1 time per week	313 (12)	1.4 (1.0-2.1)	1.1 (0.7-1.8)
				
Agrees that soldier needs	Yes	902 (9)	1 (Referent)	1 (Referent)
good physical fitness	No	487 (9)	1.1 (0.7-1.5)	0.8 (0.5-1.1)
				
Sweating exercise	≥ 3 times per week	438 (6)	1 (Referent)	1 (Referent)
(Brisk leisure time sport)	1-2 times per week	415 (8)	1.4 (0.8-3.8)	0.9 (0.5-1.6)
	Only leisured exercise	257 (12)	**2.2 (1.3-3.8)**	1.2 (0.7-2.1)
	No physical exercise	278 (15)	**2.7 (1.7-4.5)**	1.2 (0.7-2.2)
				
Participates in individual	Yes, at least sometimes	954 (9)	1 (Referent)	1 (Referent)
aerobic sports	No	431 (10)	1.2 (0.8-1.7)	0.9 (0.6-1.3)
				
Belongs to a sports club	Yes, active member	206 (2)	1 (Referent)	1 (Referent)
	No, but former member	802 (9)	**4.9 (1.8-13.4)**	**3.7 (1.5-16.0)**
	No, never member	375 (14)	**7.4 (2.7-20.4)**	**4.9 (1.2-11.6)**
				
Participates in	Yes	180 (4)	1 (Referent)	1 (Referent)
competitive sports	No	1206 (10)	**2.7 (1.3-5.8)**	1.0 (0.4-2.5)
				
Last degree in school	Good or excellent	1101 (8)	1 (Referent)	1 (Referent)
Sports	Poor or fair	286 (14)	**1.8 (1.2-2.5)**	0.9 (0.5-1.4)
				
Participates in ball games	Yes	950 (8)	1 (Referent)	1 (Referent)
	No	438 (13)	**1.7 (1.2-2.4)**	1.2 (0.8-1.8)

Variable distribution was charted in 1411 male conscripts during the first week of military service and discharge outcomes were registered during the following 6-month military service. Statistically significant findings are indicated with bold type.

* Adjusted for age (univariate)

** Adjusted for age, company, smoking (previous or current smoker), alcohol intake, baseline medical conditions (sports injury during last month, sum factor of earlier musculoskeletal symptoms during the last month before entering the military, chronic impairment or disability due to prior musculoskeletal injury, chronic disease, regular medication), school success (educational level and grades combined), urbanisation level of the place of residence, participating in ball games, last degree achieved in school sports, physical activity during the previous 3 months before entering the military, self-assessed health, belonging to a sports club and participation in competitive sports (17 adjusting variables).

### Statistical analysis

SPSS 17.0 for Windows software (SPSS Inc., Chicago, IL) was used for statistical analysis. Medical discharge incidence was calculated by dividing the number of discharged conscripts by the total number of conscripts and expressed as a percentage. Incidence rate was calculated by dividing the number of discharged conscripts by the exposure time. Exposure time was calculated until the end of the follow-up. The incidence with 95% confidence interval (CI) was expressed per 1000 person-days.

Cox's proportional hazard models were applied to study the prospective associations between baseline characteristics and discharge incidence. The outcome was defined as an incidence of premature discharge due to medical reasons. In the first phase of the Cox regression, each independent variable was analyzed one at a time. Results are expressed as hazard ratios (HR) and calculated with 95% CIs with age at baseline forced into the model.

A multivariate Cox regression was used to identify independent risk factors for discharge and examine interactions between risk factors. In the data analysis, based on the previous literature, conceptually compatible and logical risk factors were chosen for multivariate-models. Only possibly significant explanatory variables (*P *< 0.20) in the initial age-adjusted models were included for the multivariate models: Higher age, company, smoking status (previous or current regular smoker), high alcohol intake, poor baseline medical condition (sports injury during last month, sum factor of earlier musculoskeletal symptoms during the last month before entering the military, chronic impairment or disability due to prior musculoskeletal injury, chronic disease, regular medication), poor school success (educational level and grades combined) and poor self-assessed health, were entered into the model as known or possible risk factors. Prior physical activity during the previous three months before entering the military, participating in ball games, last degree achieved in school sports, belonging to a sports club, participation in competitive sports and urbanisation level of the home residence were considered as effect modifiers and entered into the multivariate model. A *P *value of less than 0.05 was considered statistically significant when interpreting the results from Cox's proportional hazard models.

## Results

### Incidence and reasons for discharge

Of the 1411 participants, 9.4% (n = 133) sustained a premature medical discharge after the 2-week run-in period during the 6-month service. The mean follow-up time per conscript was 166 days. The incidence rate for discharge was 0.57 (95% CI: 0.48-0.67) per 1000 person-days. The discharge incidence for the first (8%), second (8%), third (16%), and fourth (10%) cohorts was significantly different among cohorts (*P *= 0.002). In addition, there was a trend towards more medical discharges among arrivals entering the military in July (11%) than in January (8%; *P *= 0.058). The most common reasons for discharge were musculoskeletal injuries and disorders (44%, n = 59), followed by mental and behavioural disorders (29%, n = 39) (Table 2). For discharged conscripts, the mean time in military service (± SD) was 65 ± 37 days.

Tables 3, 4, 5, and 6 show the distribution of variables and the hazard ratios of medical discharge for various *socioeconomic *(Table 3), *health *(Table 4), *health behaviour *(Table 5), and *physical fitness *variables (Table 6) in the age-adjusted and multivariate models.

**Table 6:** Hazard ratios (HR) for early medical discharge from military service by physical fitness test variables at baseline.

Physical fitness test result	Category	Total number (% of discharged)	HR for discharge (n = 133) *	HR for discharge (n = 133) **
Cooper's test (12-minute running test)	Excellent (≥ 3000 m)	51 (6)		
			} 1 (Referent)	} 1 (Referent)
	Good (≥ 2600 m)	330 (4)		
	Fair (≥ 2200 m)	630 (6)	1.5 (0.8-2.8)	1.4 (0.8-2.7)
	Poor (< 2200 m)	358 (14)	**3.7 (2.1-6.7)**	**3.3 (1.7-6.4)**
				
Pull-up test (consecutive repeats without time limit)	Excellent (≥ 14)	158 (5)	1 (Referent)	1 (Referent)
	Good (≥ 10)	221 (8)	1.6 (0.7-3.6)	1.8 (0.7-4.5)
	Fair (≥ 6)	391 (5)	1.0 (0.5-2.4)	1.0 (0.4-2.5)
	Poor (< 6)	608 (11)	**2.2 (1.1-4.6)**	2.0 (0.9-4.6)
				
Standing long jump test (two attemps, best result observed)	Excellent (≥ 2, 40 m)	241 (5)	1 (Referent)	1 (Referent)
	Good (≥ 2, 20 m)	363 (8)	1.6 (0.8-3.0)	1.5 (0.8-3.0)
	Fair (≥ 2, 00 m)	442 (6)	1.2 (0.6-2.3)	1.0 (0.5-2.0)
	Poor (< 2, 00 m)	332 (11)	**2.3 (1.2-4.2)**	1.7 (0.9-3.3)
				
Sit-up test (repeats per 60 seconds)	Excellent (≥ 48)	221 (5)	1 (Referent)	1 (Referent)
	Good (≥ 40)	319 (4)	0.9 (0.4-2.1)	0.7 (0.3-1.7)
	Fair (≥ 32)	459 (9)	2.0 (1.0-3.9)	1.4 (0.7-3.0)
	Poor (< 32)	379 (12)	**2.8 (1.4-5.5)**	1.9 (0.9-4.0)
				
Push-up test (repeats per 60 seconds)	Excellent (≥ 38)	450 (6)	1 (Referent)	1 (Referent)
	Good (≥ 30)	312 (5)	1.0 (0.5-1.8)	0.9 (0.5-1.6)
	Fair (≥ 22)	350 (7)	1.3 (0.8-2.3)	1.0 (0.6-1.9)
	Poor (< 22)	266 (15)	**2.7 (1.7-4.5)**	**1.8 (1.0-3.2)**
				
Back lift test (repeats per 60 seconds)	Excellent (≥ 60)	660 (6)	1 (Referent)	1 (Referent)
	Good (≥ 50)	284 (10)	1.7 (1.1-2.8)	1.2 (0.7-1.9)
	Fair (≥ 40)	291 (7)	1.2 (0.7-2.0)	0.9 (0.5-1.5)
	Poor (< 40)	143 (13)	**2.2 (1.3-3.9)**	1.3 (0.7-2.4)
				
Conscript's physical fitness index ^1^	Excellent (≥ 21.00)	69 (3)	1 (Referent)	1 (Referent)
	Good (17.00-20.99)	409 (6)	2.0 (0.5-8.4)	1.4 (0.3-5.9)
	Fair (13.00-16.99)	590 (6)	2.1 (0.5-8.7)	1.1 (0.2-4.7)
	Poor (< 13.00)	297 (14)	**5.1 (1.2-21.2)**	2.5 (0.6-11.1)
				
Co-impairment in Cooper's and push-up tests	No	1219 (6)	1 (Referent)	1 (Referent)
	Yes, poor results in both tests	146 (18)	**3.1 (2.0-4.8)**	**2.6 (1.6-4.3)**
				
Co-impairment in Cooper's and pull-up tests	No	1365 (7)	1 (Referent)	1 (Referent)
	Yes, poor results in both tests	272 (15)	**2.8 (1.9-4.1)**	**2.7 (1.7-4.3)**
				
Co-impairment in sit-up and pull-up tests	No	1107 (6)	1 (Referent)	1 (Referent)
	Yes, poor results in both tests	271 (15)	**2.6 (1.8-3.8)**	**2.2 (1.4-3.4)**
				
Co-impairment in push-up and standing long jump tests	No	1241 (7)	1 (Referent)	1 (Referent)
	Yes, poor results in both tests	137 (19)	**3.1 (2.0-4.8)**	**2.5 (1.5-4.1)**
				
Co-impairment in sit-up and push-up tests	No	1215 (7)	1 (Referent)	1 (Referent)
	Yes, poor results in both tests	163 (18)	**3.0 (2.0-4.6)**	**2.6 (1.6-4.1)**

Variable distribution was charted in 1411 male conscripts during the first week of military service and discharge outcomes were registered during the following 6-month military service. Statistically significant findings are indicated with bold type.

^1 ^Conscript's physical fitness index (CPFI) = (12-min running test result (m) + 100 × muscle fitness test points)/200.

* Adjusted for age (univariate)

** Adjusted for age, company, smoking (previous or current smoker), alcohol intake, baseline medical conditions (sports injury during last month, sum factor of earlier musculoskeletal symptoms during the last month before entering the military, chronic impairment or disability due to prior musculoskeletal injury, chronic disease, regular medication), school success (educational level and grades combined), urbanisation level of the place of residence, participating in ball games, last degree achieved in school sports, physical activity during the previous 3 months before entering the military, self-assessed health, belonging to a sports club and participation in competitive sports (17 adjusting variables).

From the *socioeconomic background *variables (Table 3), a conscript's poor school success (educational level and degrees combined) was the strongest risk factor. After adjustment in multivariate analyses, poor school success was associated with a 4.6-fold higher risk for discharge (95% CI: 2.0-11.0) compared to excellent school success with a graded relationship. Older age was associated with discharge in the age-adjusted model, but was not significant in multivariate model.

With regard to *health *(Table 4), we observed low self-assessed health to be the strongest risk indicator in a graded manner (HR 2.8; 95% CI: 1.6-5.2) after adjustments in multivariate analyses. Waist circumference over 102 cm was clearly associated with discharge compared to normal waist circumference. In addition, chronic diseases and former sport injuries were associated with discharge.

From the *health behaviour *variables (Table 5), never belonging to a sports club was a strong risk indicator for discharge (HR = 4.9; 95% CI: 1.2-11.6). Conscripts who used alcohol more than once a month seemed to have lower risk for discharge compared to conscripts who drank alcohol less frequently. Smoking and lack of participation in leisure time sports before entering military service were associated with discharge in the age-adjusted model, but these associations weakened in the multivariate analyses.

With regard to *physical fitness *(Table 6), we observed a clear association between low physical fitness and discharge. In the age-adjusted analysis, all the army physical fitness tests were associated with premature discharge. After adjustment in the multivariate analyses, the strongest association was between a poor result in the 12-min running test and discharge (HR = 3.3; 95% CI: 1.7-6.4). In addition, a poor result in the push-up test nearly doubled the risk for discharge. When combining individual fitness test results, co-impairment in 12-min running and push-up or pull-up tests was the strongest risk indicator. In addition, co-impairments in sit-ups, push-ups, pull-ups, and standing long jump test were associated with discharge.

There were some associations for risk factors specific for mental or musculoskeletal discharge categories (Table 7). Low self-assessed health was associated especially with discharge for mental reasons (HR = 7.8; 95% CI: 2.7-22.4). Use of alcohol more than once per month was associated with a lower risk for discharge due to mental reasons. Co-impairment in the sit-up and push-up tests was associated especially with discharge for musculoskeletal reasons. Older age was associated with discharge for mental reasons. There was a trend towards poor school success being associated with discharge for mental reasons.

**Table 7:** Hazard ratios (HR) for early medical discharge stratified by musculoskeletal and mental reason categories.

Variable	Category	Total number (% of discharged^§^)	HR for discharge^§^ (n = 133) *	HR for discharge^§^ (n = 133)
Discharge due to musculoskeletal reasons				
				
Urbanisation level of the place of residence	< 10000 inhabitants	537 (3)	1 (Referent)	1 (Referent) ^†^
	≥ 10000 inhabitants	850 (5)	**1.9 (1.1-3-4)**	**2.3 (1.3-4.4) **^†^
				
Chronic disease	No	1012 (4)	1 (Referent)	1 (Referent) ^†^
	Yes	377 (6)	1.6 (1.0-2.8)	**1.8 (1.0-3.2) **^†^
				
Co-impairment in sit-up and push-up test	No	1215 (3)	1 (Referent)	1 (Referent) ^†^
	Yes, poor results in both tests	163 (7)	**2.6 (1.4-5.1)**	**2.4 (1.2-4.7) **^†^
				
Discharge due to mental reasons				
				
Age	18-19 years	1052 (2)	1 (Referent)	1 (Referent) ^‡^
	20-28 years	359 (5)	**2.9 (1.5-5.4)**	**2.7 (1.4-5.3) **^‡^
				
Self-assessed health ^1^	Good or very good	743 (1)	1 (Referent)	1 (Referent) ^‡^
	Average	558 (3)	**3.0 (1.3-6.9)**	2.1 (0.9-5.4) ^‡^
	Inferior	88 (15)	**15.4 (6.4-37.2)**	**7.8 (2.7-22.4) **^‡^
				
Use of alcohol	< 1 time per month	254 (5)	1 (Referent)	1 (Referent) ^‡^
	1-2 times per week	894 (1)	**0.3 (0.1-0.7)**	**0.3 (0.1-0.6) **^‡^
	≥ 3 times per week	240 (5)	1.1 (0.5-2.5)	0.6 (0.3-1.4) ^‡^

Variable distribution was charted in 1411 male conscripts during the first two weeks of military service and discharge outcomes were registered during the following 6-month military service. Statistically significant findings are indicated with bold type.

^§ ^Discharge due to musculoskeletal or mental reasons

^1 ^Compared to age-mates

* Adjusted for age (univariate)

^† ^Adjusted for age, company, father's occupational group, smoking (previous or current smoker), frequency of drunkenness, baseline medical conditions (sum factor of earlier musculoskeletal symptoms during the last month before entering the military, chronic disease), school success (educational level and grades combined), urbanisation level of the place of residence, participating in ball games, last degree achieved in school sports, physical activity during the previous 3 months before entering the military, self-assessed health, belonging to a sports club and participation in competitive sports (15 adjusting variables).

^‡ ^Adjusted for age, alcohol intake, baseline medical conditions (sum factor of earlier musculoskeletal symptoms during the last month before entering the military, chronic impairment or disability) school success (educational level and grades combined), participating in ball games, last degree achieved in school sports, physical activity during the previous 3 months before entering the military, self-assessed health and participation in competitive sports (10 adjusting variables).

## Discussion

Low levels of physical fitness, poor school success, poor self-assessed health, and high waist circumference were associated with premature discharge from military service in a graded manner. Conscripts that never belonged to a sports club were at higher risk of discharge compared to former club members and especially present active members. Of the 1411 participants, 9.4% (n = 133) sustained premature medical discharge during the 6-month service. The most common reasons for discharge were musculoskeletal (44%, n = 59) injuries, followed by mental and behavioural disorders (29%, n = 39). The hypothesis that co-impairment in physical fitness is a predictor of medical discharge was based on our previous study investigating risk factors of musculoskeletal disorders during military training [9].

Santtila and colleagues [24] reported that conscripts' aerobic fitness has decreased 12% during the years 1979-2004 and mean body mass has increased 4.4 kg during the years 1993-2004. Moreover, the proportion of conscripts with low physical ability leading to problems meeting minimum physical requirements set for military service has increased dramatically: The number of conscripts with a poor result (< 2200 m) in Cooper's test increased 5.6-fold between 1980 and 2004 [24]. Poor muscle fitness and aerobic capacity [9,25-28] and obesity [9,25,29] are risk factors for musculoskeletal injuries and disorders among conscripts. Conscripts' tasks requiring both strength and aerobic capacity, such as loaded marching, may be further negatively affected by obesity [24], demonstrating a situation where several components may play an important role in the aetiology of musculoskeletal injury. In the present study, high waist circumference was independently associated with premature discharge compared to normal waist circumference, whereas BMI was not. This was probably due to the fact that BMI does not distinguish lean mass from fat tissue [30].

One of the reasons for the current study was that at the turn of the millennium, there was a substantial rise (62%) in the number of premature discharges in the Finnish army due to musculoskeletal injuries [31]. Most likely, this was due to the 100% increase in physical exercise in the Finnish military service program in July 1998. At that time, 8% to 10% of the conscripts were prematurely discharged from the Finnish Defence Forces. In a recent study, we found that co-impairments in cardiorespiratory and muscular fitness (i.e., poor results in Cooper's test combined with a poor result in standing long jump, push-up or back lift tests) were highly associated with musculoskeletal injuries and disorders, showing a dose-response relationship. Similarly, abdominal obesity and high BMI were clearly associated with the outcome [9].

Belonging to a sports club is strongly associated with leisure time physical activity, which seems to lower the risk for discharge [12,14]. Sports clubs may also enhance health in ways other than through physical fitness. Koski [32] reported that 81% of Finnish youth sports clubs declare that healthy lifestyle is one of their main goals. Moreover, sports clubs offer informal education on teamwork, interaction skills, and assessing values [33]. Other factors associated with benefits acquired in sports clubs may be that in sports clubs children and adolescents learn to obey rules and follow the instructions of coaches, skills that probably help conscripts to adapt to the discipline required for compulsory military service.

The present results indicated that poor self-assessed health predicted discharge especially for mental health reasons. Similar findings have been reported among Swedish conscripts [14] and US Air Force recruits [34]. Multimaki et al. [1] also found that mental health service use was strongly associated with medical discharge at call-up. In a recent Finnish study, psychosocial problems were more prevalent among men who interrupted their service compared with those exempted from service at call-up [19]. This can be explained by the fact that somatic diseases can be identified more easily than psychosocial problems at call-up. Based on the present findings, direct questions about mental and physical well-being can be used to distinguish persons with an elevated risk for discharge before the onset of military training. Moreover, mental reasons leading to discharge tend to be long-term and debilitating. Only every seventh conscript discharged due to mental reasons performs military service in a 5-year follow-up after the discharge [16].

Our results showed that conscripts who used alcohol more than once a month had a lower risk for premature discharge, especially for mental health issues. This may be due to anxiolytic effects of alcohol during vacations from military service. Andreasson et al. [35] supported this hypothesis and concluded that conscripts who were never anxious or never felt insecure used more alcohol than their counterparts. In contrast, however, Ristkari et al. [36] reported that a high level of alcohol use was associated with poor coping and resiliency strategies among young men at military call-up [36] and excessive alcohol use is associated with discharge at call-up [1]. Another possible explanation for our contradictory finding might be that regular use of alcohol is seen as normal behaviour for conscripts during vacations and this improves affinity among conscripts who use alcohol [37].

The present study has several strengths. First, the definition of premature discharge due to medical reasons was clear and defined by ICD-10 codes set by an independent physician in the garrison clinic. Second, the garrison clinic computerised patient records were cross-checked with the discharge data of the Finnish Defence Forces, guaranteeing a high coverage of discharges. Third, the participation rate was high (98%). Fourth, the military environment provides highly standardised conditions for investigating the effect of individual risk factors: conscripts underwent daily military programs that were nearly equal, providing equal opportunity for rest and sleep [26]. Given that 90% of young men in Finland enter military service, the present results regarding musculoskeletal injuries and disorders might have an impact also outside military environment among young males who engage in an intensive physical training program with different physical fitness, body characteristics, health behaviour, and socioeconomic backgrounds.

Our study has also limitations. First, although the compulsory military service concerns all Finnish male citizens, approximately 15% of conscripts are exempted from duty after physician examinations at call-up or during the first week of military service due to minimum physical and mental requirements established for military service [2]. Second, approximately 7% of all eligible men choose to perform non-military service in Finland [38]. Third, although the information of waist circumference length was available in 93% of conscripts, it was missing in over 30% of discharged conscripts because they were exempted from active service due to flu or musculoskeletal injuries when the waist circumference was measured. Hence the variable was not entered into the adjusted model which is a limitation of the study. Fourth, the findings can be generalized to young men only because no more than 3% of the conscripts were females and they were excluded from the study. A fifth limitation was the fact that after the initial 8 weeks of basic training, the training programs became more divergent due to the more specialised military service in each company. This also caused drop-out of some participants due to a company change. On the other hand, all conscripts were followed up for the first 8 weeks of service and results were adjusted by company.

## Conclusion

In Finland, 13% to 15% (3500-4000 persons) of young men who enter the military service are prematurely discharged annually from compulsory military service. In the present study, low levels of aerobic and muscular fitness and poor school success were associated with premature discharge from military service in a graded manner. We also found that poor self-assessed health was especially associated with discharges due to mental health reasons. These findings highlight the need for an improved pre-enlistment examination. The new interesting finding was that conscripts who had never been a member of a sports club had an elevated risk for premature discharge. For the conscript, a premature discharge during military service can cause financial, emotional, and physical harm requiring long-term rehabilitation. Discharged conscripts are at risk of being marginalised in society at a time when they are at the threshold of adulthood [1,19]. Especially mental health reasons leading to discharge are associated with poor income, retirement, divorced or single status, and a criminal record [39,40] in a follow-up of 10 to 23 years after compulsory military service. Preventive measures and programs are clearly needed and, optimally, should be tested in controlled intervention studies. The present findings suggest that increasing both aerobic and muscular fitness is a desirable goal in a pre-training program before entering military service. Attention to appropriate waist circumference and strategies addressing psychological well-being may strengthen the preventive program.

## Competing interests

The authors declare that they have no competing interests.

## Authors' contributions

HT wrote the first draft of the manuscript together with AJMH. HT and AJMH also participated in data analysis, interpretation and data acquisition. JHS was the primary investigator together with JP. JHS initiated and conceptually designed the study and took part in data processing and manuscript reviewing. HP participated in study concept and design as well as manuscript reviewing. JP initiated and conceptually designed the study and participated in manuscript writing, data analysis and interpretation. All authors have made substantive intellectual contributions to the study. All authors reviewed the article and gave the final approval of the manuscript.

## Pre-publication history

The pre-publication history for this paper can be accessed here:

http://www.biomedcentral.com/1471-2458/11/590/prepub
